# Self-quarantined: becoming accustomed to being a semi-prisoner

**DOI:** 10.3325/cmj.2020.61.386

**Published:** 2020-08

**Authors:** Charles H. Calisher

This coronavirus pandemic is not too bad a situation for me. No one in our family is dead, sick, or even bored. Indeed, we have been very busy since beginning self-quarantining in about mid-March. I am retired from the university now, so am busy each day writing, editing, consulting, annoying editors, and pretending that today will be different from yesterday. It is like being in a prison without bars. In order to keep from losing what remains of my mind, I have tried a variety of methods.

1. I turn on the television each morning to see the pandemic up-date. Mostly, it is a repeat of yesterday’s news, except that the numbers of people with diagnosed COVID-19 world-wide increase each day, as do the numbers of those dead from this terrible pandemic disease. I am sad for those whose loved ones have died or are in critical condition but pleased that neither I nor my loved ones have been stricken so far. (We might take this opportunity to not tell certain people when all this ends, assuming, of course, that it will end. You can make your own list of who should be informed that the danger has passed, so they can go outdoors without a mask again. Those not on your list would be on their own. I am not concerned about crowds in the streets and predictions of the end-of-the-world; this is democracy in action.)

2. I eat breakfast and then check my e-mails, in case there is something I need to do right away. There always is, so I do it. Then I go outside to the garden, which is progressing slowly but methodically and very nicely, being that Spring has arrived in the Rocky Mountains. Strange weather being another situation to which we have become accustomed, in Colorado it goes from hot to cool, wet to very dry, and insect-free to being inundated with migrating miller moths (the adult stage of the “army cutworm” [*Euxoa auxiliaris*], which damage a variety of spring crops). I know it is a sign of desperation when I spend time reading about arthropods that are not known to transmit viruses to humans.

3. After I have checked the iris plants [order Asparagales; that is all anyone needs to know, unless someone is paying you to know more] and seeing that they have not bloomed, I go back in the house and rest.

4. If I do not fall asleep, I check the television again to see whether the state government has re-opened things a bit. Among our other peculiarities, in the U.S.A. individual states have certain constitutional “rights” that exceed rights assigned to the federal government, so situations can be and usually are different from those in various states, county rights may differ within states, and cities have some independence. In my home, we have different rights from anyone else’s. For example, I can make a telephone call while naked, which I cannot legally do outside my home. They can relax state mandates if they want but I plan to stay right here until I see what happens to my neighbors when they go out to celebrate. Some say that the epidemic curve is “flattening,” so we can begin lifting restrictions, but that seems analogous to a person recognizing that a parachute slowing one’s rate of descent is sufficient evidence to safely remove it. I will just wait a bit more. Having a “flat” epidemic curve of 800/d is not, to me, a consolation.

5. Some have questioned whether this newly recognized coronavirus is REALLY all that serious. Let me put it this way: churches, synagogues, and mosques, as well as casinos, are closed. When heaven and hell agree on the same thing, it is probably a serious situation.

6. Our new monthly budget is: petrol $0, entertainment $0, clothes $0, food $2000. I stepped on my bathroom scale this morning and it said: “Please practice social distancing. Only one person at a time on the scale.”

7. I am proud to say that I have not been late for a meeting since about mid-March, but I probably should not take credit for this.

8. This pandemic has turned us into dogs. We wander around the house looking for food, we get told “No,” if we get too close to strangers, and we become excited about going for walks and rides in the car.

9. When I am not in the garden, I am writing and reading a great deal; mostly reading, however. News magazines, college alumni bulletins, natural history publications, labels on food packaging, and travel brochures (Mars seems to be nice at this time of year). There is grass to cut, weeds to kill, plants to water, bushes to trim, tools to store, and a grill to clean. I feel very badly about leaving all this to my wife but the exercise alone keeps her healthy and in good physical shape. I began reading “War and Peace” in March but expect the pandemic will end before I arrive at the last page.

10. One day I sneaked out to go to a grocery store in a small village near our house. I did not have a mask with me so, when I arrived there, I tied a bandana (c.f. *rubac*) on my face, did a little shopping, and added myself to the line leading to the cash register. By the time I had made my way to the cashier, there were about six people behind me, all wearing face masks. The cashier smiled, said “Hello” and tried to hand me some money from the cash register. I asked what that was for and he said, “I thought this was a robbery.” It is good, even necessary, to maintain a sense of humor during a quarantine.

11. I am thankful that our children are old enough not to be home-schooled during this situation, let alone no longer living here. Having taught reading to 8-year-olds for five years at a local school, I cannot imagine doing that now. It may “take a village to raise a child,” but it might take a vineyard to home-school one.

12. This afternoon I checked the garden again; no iris blooms yet.

13. I made a list of chores to do in our house, ie, oil squeaky doors, tighten loose screws, replace dead light bulbs, kill the miller moths that prefer living with us to living outside where their predators are waiting, organize my desk, organize my tools, and find my 5-year-old “to do” list. Nonetheless, I have not had time to do any of this.

14. We went for a drive the other evening. At first, we considered going to Santa Fe, New Mexico (730 km one way) to find a restaurant (great Mexican food in Santa Fe) but that seemed to be a long way to go only to find restaurants closed there. So, we drove around our home town and found closed restaurants more conveniently located. Plenty of parking spaces also. Even the police are bored, or so they have told me.

15. Being an enthusiastic sports fan, I pay attention to what is going on, but nothing is going on. Baseball, American football, basketball, ice hockey, European football, and more, are seasonal sports. However, none are pandemic season sports, so the various television stations are replaying video versions of “great games” from the past. My problem with this wonderful idea is that (a) I am not interested in who wins and who loses in games that were played 15 years ago and, anyway, I did not watch them at that time either and (b) watching repetition of poor play is worse than watching it for the first time. I am more interested in tomorrow than in yesterday, which I already know about.

16. I have not worn a wristwatch since some time in April. It’s difficult enough to remember what day it is.

17. I thought about renewing my driver’s license (it does not expire for another two years), recycled all our aluminum cans, cleaned the patio twice (even though it was clean before the second time I did it), corrected the spelling of “mayonnaise” on a jar label, and am paying attention to the inexorable and sorrowful increases in deaths, here and everywhere else. Distressingly, I am becoming accustomed to it all.

18. The only good outcome of this pandemic situation that I can see is that it predicts job security for virologists and many others.

19. As of this evening, the irises have not yet bloomed – perhaps tomorrow.

